# International Undergraduate Students in Chinese Higher Education: An Engagement Typology and Associated Factors

**DOI:** 10.3389/fpsyg.2021.680392

**Published:** 2021-06-23

**Authors:** Mei Tian, Genshu Lu, Lijie Li, Hongbiao Yin

**Affiliations:** ^1^School of Foreign Studies, Xi'an Jiaotong University, Xi'an, China; ^2^School of Humanities and Social Science & West China Higher Education Evaluation Center, Xi'an Jiaotong University, Xi'an, China; ^3^School of Public Policy and Administration, Xi'an Jiaotong University, Xi'an, China; ^4^Faculty of Education, The Chinese University of Hong Kong, Hong Kong, China

**Keywords:** international students, student engagement typology, individual differences, learning environment, China

## Abstract

This research adopted a typological approach to explore international students' academic engagement in China. Using data generated by a survey study involving 801 international undergraduate students at 34 full-time Chinese universities, this research developed an international student engagement typology, and examined important individual and learning environment factors associated with the engagement types presented in the typology. The international student engagement typology helps to understand and enhance international undergraduate students' learning experiences in Chinese HEIs. Although located in China, this research holds implications for practitioners in broader contexts striving for the sustainable development of international student education.

## Introduction

China is the largest international student source country globally, but it has been enhancing its attractiveness to inwardly mobile students over the past decade. The significant increase in the number and diversity of international students studying in Chinese Higher Education institutions (HEIs) led to a growing number of studies on these student experiences (Tian et al., 2020). An exploratory interview study (Tian and Lowe, 2018) showed that the major reason for international degree students' decision to study in China was to gain a qualification that would lead to employment, whereas it was the education they received, rather than their social or cultural experiences, that attracted these students' most complaints. Other research on international students in China consistently reported low levels of academic satisfaction (Haugen, 2013; Ding, 2016), language barriers (Wen et al., 2018) and insufficient teacher-student interaction (ibid). The findings have raised prominent concerns in the academic and policy discourse over Chinese universities' capacity to monitor their international student education (Kuroda, 2014).

Worldwide, student engagement has been regarded as a valuable indicator of education quality (Coates, 2005). National surveys on college student engagement are conducted annually in America (Kuh, 2003, 2009a), Australia (Coates, 2010), and several Asian countries, such as China (Shi et al., 2014). One way to use the student engagement research data is to identify a typological model of student engagement. The typological approach differentiates college students into distinct groups based on the analysis of their engagement characteristics (Hu and McCormick, 2012). The approach allows institutions to compare the results with peer institutions or with the data from the previous years, highlighting the strength in practice and weakness for improvement.

This article reports on the development of an engagement typology of international students in Chinese HEIs and the individual and environmental factors associated with the engagement types presented in the typology. Data were generated by a survey study involving 801 international undergraduate students at 34 Chinese universities. This research is one of the first using a typological approach to investigate international undergraduate students' engagement in educational practices in China. It contributes to the understanding and enhancement of international students' learning experiences in Chinese HEIs. Although located in China, this research holds implications for practitioners striving for the sustainable development of international student education in broader contexts.

## International Students and He in China

Since the turn of the new century, with its booming economy and accelerated HE development, China has strengthened its ability to attract inward international students. Between 2004 and 2008, the number of international students studying in China grew at an average rate of over 18% annually (tsinghua.edu.cn, 2010). In 2010, China announced the National Outline for Medium and Long-term Education Reform *and Development (2010–2020)*, stressing the role of internationalization in the quality enhancement of Chinese higher education (Central Government, 2010). Following this blueprint policy, the MoE released the Study in China Scheme, the first national strategic plan on international student recruitment (Ministry of Education, China, 2010; Ross et al., 2011). In 2015, President Xi Jinping announced cooperation in education as an essential dimension of the nation's high-profile Belt and Road Initiative (yidaiyilu.gov.cn, 2015), stressing China's determination to contribute to education development in the Belt and Road region (Ministry of Education, China, 2016). In 2017, the main target of *the Study in China Sc*heme was achieved, with China becoming the largest destination country in Asia (Guangming daily, 2018). In 2018, the number of international students studying in Chinese HEIs reached 492,185 (Ministry of Education, China, 2019). Among these students, 258,122 were degree students and 173,060 were pursuing an undergraduate degree (ibid). In the same year, *the Quality Standards for International Student Higher Education in China (trial version)* was released (Ministry of Education, China, 2018), marking the development of the country's international education enters into a new stage of “improving quality and enhancing efficiency.”

One feature of Chinese international education is the rapid enrolment expansion of international undergraduate students in sciences and engineering disciplines, particularly at the “double-first-class” universities (State Council, China, 2015). The double-first-class initiative, which replaces the precedent 211- and 985- projects, provides generous national funding to the listed institutions and disciplines to achieve world-leading academic excellence (Ministry of Education, China, 2017). This initiative, supporting the building of an innovation-driven economy, has prioritized science and engineering disciplines (Liu, 2018). The resulting rapid enhancement in academic strength (Shanghai ranking, 2019a,b) has turned these “hard disciplines” into popular choices for international students. Engineering, for example, rose from the 10th most popular major for international undergraduate students in China in 2010 (Ministry of Education, China, 2011) to the 2nd in 2018, following medicine only (Ministry of Education, China, 2019).

## Student Engagement

With the growing stress on assessment, transparency and accountability, student engagement has been widely accepted as a valid indicator of institutional performance and has featured widely in the discussions of HE quality (Kuh, 2009a). One widely cited definition of student engagement is “the time and effort students devote to activities that are empirically linked to desired outcomes of college and what institutions do to induce students to participate in these activities” (Kuh, 2009b, p. 685). This view of student engagement reflects the constructionist idea that students exert agential power to construct their knowledge through participating in educational activities (Coates, 2005). It, in line with the self-determination theory, proposes that individual learners, through active engagement with contextual challenges and facilitators, fulfill their potential capacity for learning (Reeve, 2012).

The constructionist view of student engagement forms the basis of the National Survey of Student Engagement (NSSE), an annual nationwide survey involving public and private HEIs in the United States. NSSE interprets student engagement as a multi-faceted concept, consisting of active and collaborative learning, academic challenge, student-faculty interaction, enriching educational experience and supportive campus environment (Kuh, 2003). Following this interpretation, a large amount of research has been conducted. Research findings reported on the relationship between student engagement and desirable college outcomes, as represented by reduced dropout rates (Kuh et al., 2008), improved grades (Rissanen, 2018), gains in general abilities (Pike et al., 2003) and intellectual development (Hu et al., 2008).

To further understand the complexity of student engagement, several typological models have been developed. One early typological analysis was performed by Astin (1993). Using the American national survey data from the Cooperative Institutional Research Program, the analysis generated seven engagement-based student types- scholar, social activist, artist, hedonist, leader, status striver, and uncommitted. Another influential typological work was conducted by Kuh et al. (2000). The investigation used the national data that were generated from the College Student Experience Questionnaire (CSEQ). Analysis of student engagement in meaningful educational activities resulted in 10 student types, i.e., intellectual, grind, scientist, collegiate, recreator, socialiser, conventional, individualist, artist, and disengaged.

More recently, Coates (2007), based on survey data from 1,051 undergraduate students in Australia, proposed four types of student engagement, i.e., intense, collaborative, independent, and passive. Intense engagement was characterized by active participation in rich learning activities, peer collaborations and communication with faculty. Independent engagement involved an individualistic, academic-oriented approach to learning. Collaborative engagement stressed social, rather than cognitive aspects of engagement. Passive engagement involved rare participation in quality learning activities. The research revealed the importance of the student engagement typological approach in college quality assurance, with its values to assess student engagement type distribution in comparison with peer institutions (Hu and McCormick, 2012).

**Figure 1:**
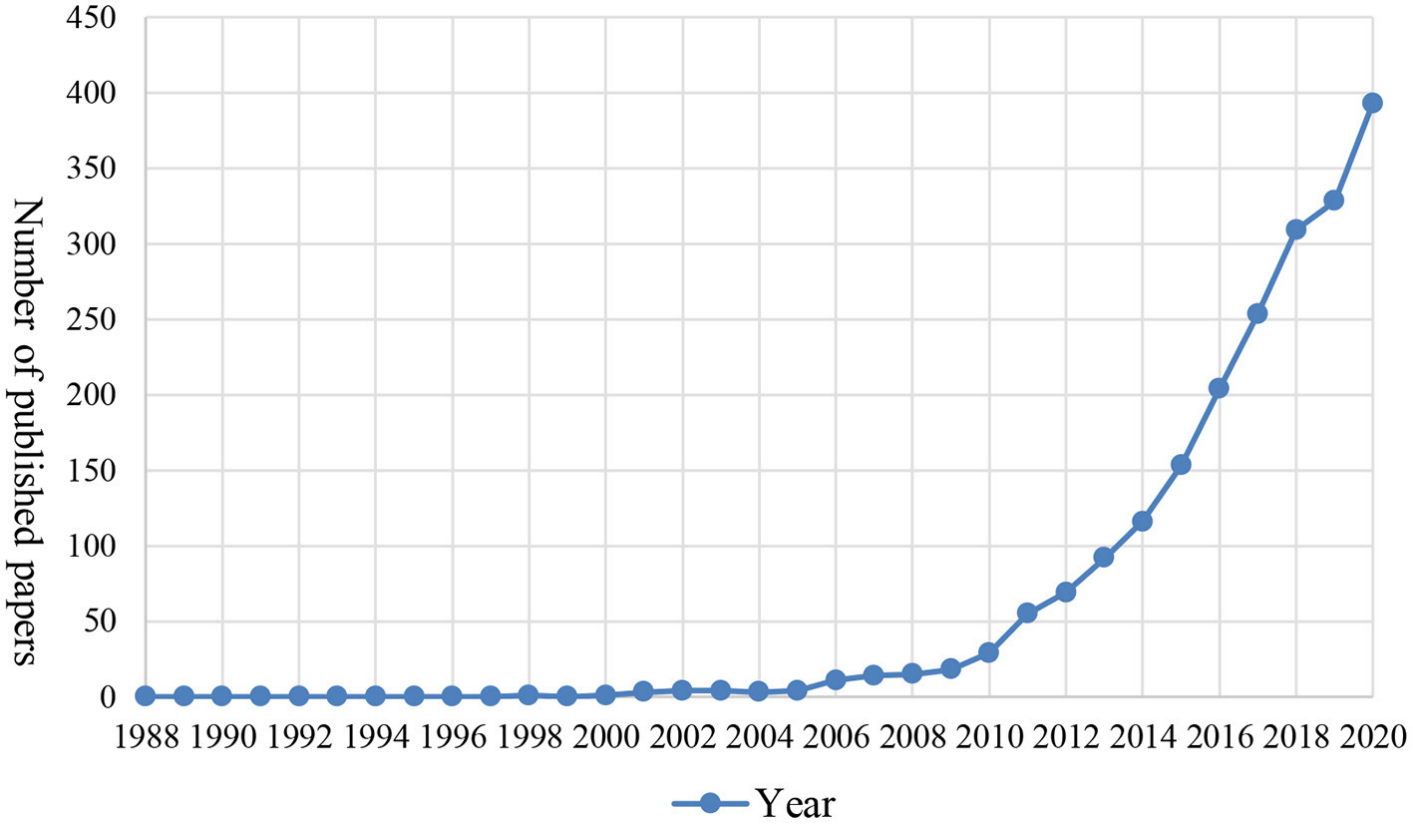
Annual distribution of Chinese domestic publications on international student engagement indexed in CNKI.

In China, student engagement was popularized by Shi and her colleagues at Tsinghua University, who modified NSSE into the China College Student Survey (Shi et al., 2014). The CCSS has aroused interests among Chinese HEIs facing competition for funding and pressure for public accountability (Ross et al., 2011), leading to emerging research on native college students' engagement in China. For the purpose of this research, the authors applied the advanced search strategy using the term “*xuesheng touru*” (student engagement) and its synonym “*xuexi touru*” (learning engagement) in the China National Knowledge Infrastructure (CNKI)^1^, the largest online database of Chinese domestic academic publications. Three thousand, five hundred fourteen academic publications were extracted, with all containing the aforementioned terms in either titles, abstracts or keywords and published between 1988 (the year in which the first publication on the topic was covered in the database) and 2020. After removing the irrelevant publications, for example those on financial investment, 2,079 valid records were finally obtained. Figure 1 presents the annual distribution of the 2,079 publications, showing a sharp growth in the number of publications related to student engagement over the recent decade.

Using simultaneously the terms “*liuxuesheng*” (international students) and “*xuesheng touru*,” or “*liuxuesheng*,” and “*xuexi touru*,” to search for the academic publications in CNKI that examined international student engagement in China, the research initially retrieved 27 publications. After removing the irrelevant publications, the research obtained 12 valid results, including seven journal articles and five Master's dissertations. Among the journal articles, five investigated, respectively, the characteristics of international students' Chinese learning engagement (Wang et al., 2015; Wang, 2017), the factors influencing their Chinese learning engagement (Wang et al., 2015; Shi and Gao, 2017; Wang, 2017), and the relationship between their Chinese learning engagement and Chinese learning outcomes (Xiong and Ding, 2014; Gu and Shi, 2016). Another two journal articles focused on general learning experiences of international students in Chinese HEIs, both of which involved some discussions on these students' participation in educational activities (Gao et al., 2019; Su, 2019). The five Master's dissertations explored, respectively, the levels of international student engagement (Yang, 2019), the variations of international student engagement across demographic variables (Liu, 2020), the mediating effects of international student engagement between their intercultural sensitivity and learning outcomes (Li, 2018), the correlation between international students' Chinese learning engagement and their Chinese learning performance (Zhang, 2019), and the relationship between international student engagement and quality of international doctoral education in China (Zong, 2015).

The research then applied the advanced search strategy in CNKI, using simultaneously “*laihua liuxuesheng*” (international students in China) and “*xuesheng touru*,” or “*laihua liuxuesheng*” and “*xuexi touru*,” and retrieved four relevant publications, all of which have been reviewed above (i.e., Zong, 2015; Shi and Gao, 2017; Li, 2018; Liu, 2020). The research retrieved no relevant results in CNKI, using simultaneously the terms “*laihua liuxue*” (study in China) and “*xuesheng touru*,” or “*laihua liuxue*,” and “*xuexi touru*” as searching keywords. Similarly, the research obtained no relevant results, using simultaneously “*waiguo xuesheng*” (foreign students) or “*haiwai xuesheng*” (overseas students), and “*xuesheng touru*” or “*xuexi touru*” as searching keywords.

Although only covering the Chinese literature indexed in CNKI, the review evidences that Chinese domestic studies on the engagement of international students in Chinese HEIs remain very limited, of which, to the best of our knowledge, none has adopted a typological approach. The engagement-based quality evaluation research and the prompt feedback it provides are critical to the sustainable development of Chinese international education with the rapid expansion of the international student population in China. Therefore, it is of importance to further investigate international student engagement in Chinese HEIs through the use of the typological approach and explore the factors associated with the types of international student engagement presented in the typology.

## Factors Associated With Student Engagement

### Individual Differences in Student Engagement

Given the significance of engagement to student learning (Kuh et al., 1991), it is of importance for institutions and practitioners to monitor engagement patterns systematically and explore whether and how engagement varies among students' demographic subgroups (Krause and Coates, 2008). With regard to gender differences, much research has been conducted. For example, Kinzie et al. (2007) compared engagement patterns of 472,985 male and female undergraduates at 487 American and Canadian HEIs. Descriptive statistics and hierarchical linear modeling revealed that female participants displayed higher levels of engagement in academic studies than their male counterparts. Other American researchers reported that female students in colleges were more likely than males to attend class on time (Pryor et al., 2007), spend time on study (Pryor et al., 2007), participate in extracurricular learning activities (Freeman, 2004) and have frequent and meaningful interactions with their faculty (Sax and Harper, 2005). Although major research during the 1970s reported that female undergraduates were significantly less engaged than male counterparts at historically black American colleges (Allen, 1986), more recently African American females were found to spend more time on study and work harder than males to meet academic challenges (Harper et al., 2004). In Chinese HEIs, the CCSS annual results reported that female students scored significantly higher than male peers on time invested in learning, learning autonomy, extracurricular activities, and experimental learning activities (Shi et al., 2011).

Previous studies also explored the relationship between college grade level and student engagement. Kuh (2007), for example, found that first-year students in American colleges engaged less in reading, writing and other learning activities than they had initially expected. NSSE 2020 showed that both first-year and senior-year students devoted more time to study than NSSE respondents did in 2004 (Nietzel, 2020). A case study of Tsinghua University in China revealed that upper-class students, seniors in particular, displayed significantly less academic engagement than first-year students did, with respect to their meeting of challenging course requirements (Wen et al., 2014).

In addition, much research has evidenced racial/ethnic differences in student engagement (Harwood et al., 2012), but only a few discussed the effect of international student status on engagement. Zhao et al. (2005), drawing on the NSSE 2000 dataset, compared the engagement levels of first-year international students and their American peers. The research found that the international students were more engaged in meeting academic challenges and interacting with faculty. In contrast, Grayson (2008) reported that international students had similar levels of engagement, but a lower level of academic support, when compared with those of domestic American students. Later on, Korobova and Starobin (2015), using the NSSE 2008 dataset, found that international students engaged less than native students in the activities of “writing reports,” “tutoring or teaching other students,” “participating in a community-based project,” “working with faculty members,” taking “internship” and engaging in “community service” (p. 80).

Other research suggested the influence of discipline and institution type on student engagement (Coates, 2005). The empirical analysis in the United States found that given the stress on interactions and communications in arts, humanities and social sciences, students in these disciplines reported higher levels of cooperative learning than their counterparts in sciences, engineering and agriculture (Matthews et al., 2011). It was also reported that students at liberal art colleges in America were better engaged in learning (Umbach and Wawrzynski, 2005). In China, Author reported the significant differences in critical thinking, meeting academic challenges, analytical understanding and extracurricular engagement among Chinese undergraduate students with different disciplinary backgrounds. Based on the analysis of 8-year CCSS data (2011–2018), Huang et al. (2021) reported that Chinese undergraduates in double-first-class universities scored higher on engagement in reading, writing and problem-solving activities, while students in non-double-first-class universities engaged more in collaborative learning and interaction with faculty.

### Learning Environment and Student Engagement

The constructivist conceptualisation of student engagement, while placing ultimate significance on human agents, stresses the importance of learning contexts (Krause and Coates, 2008). Students' interactions and communications with the learning environment constructed by institutions and faculty affect the nature and extent of their engagement in activities, which in turn influences their perceptions and evaluation of the quality of education. Here learning environments refers to social, pedagogical and psychological contexts in which student learning takes place and which have influences on their learning outcomes (Lim and Fraser, 2018).

Much research has explored the influences of learning environment on student engagement. Regarding classroom contexts, Umbach and Wawrzynski (2005), using American NSSE dataset, suggested that student engagement was encouraged by faculty members who stressed active and collaborative learning, integrated high-order cognitive activities in classrooms, were able to challenge student academically and had frequent course-related interactions with students. Ahlfeldt et al. (2005) reported that innovative teaching methods, which allowed students to work within groups and explore solutions to problems, better encouraged active engagement than traditional teaching methods. A survey study conducted by Almarghani and Mijatovic (2017) in Libya suggested that teachers' competencies in introducing active learning techniques played an essential role in encouraging and promoting university students' engagement. Matthews et al. (2011) reported that informal learning spaces enabling students to work collaboratively outside classes enhanced their levels of learning engagement.

Regarding campus learning environment, much research has focused on campus climate as perceived by racial, religious, ethnic, and sexual minorities (Ancis et al., 2000; Seggie and Sanford, 2010; Tetreault et al., 2013), and how their perceptions have affected learning engagement (Cabrera et al., 1999; Hurtado and Ponjuan, 2005). It has been suggested that to help “non-traditional” students to be more engaged, faculty and institutions should ensure hospitality, safety, equal power relationships, and critical awareness of these students' negative learning experiences (Mann, 2001).

Studies on the general experiences of international students, although rarely categorized as “engagement” research, consistently highlighted the significance of learning environments to intercultural learning and development. Institutions and faculty, through carefully designing courses, adopting inspiring pedagogy and providing opportunities for collaborative learning, were able to encourage international student participation in learning, which in turn supported their linguistic, social and academic adaptation (Tian and Lowe, 2013). A safe, friendly, caring, inclusive and supportive campus was crucial for fostering a stronger sense of belonging (Tian and Lowe, 2009, 2014). On the other hand, negative campus climates with intolerance or disrespect for diversity resulted in feelings of frustration, loneliness and powerlessness (Tian and Lowe, 2009, 2018). Social exclusion, prejudice and discrimination, perceived or real, could significantly affect international students' interactions with host communities and discourage further engagement with host higher education (Tian and Lowe, 2009, 2018).

Although the findings were mixed, the research provided evidence that engagement levels were likely to differ by individual demographics. Previous studies also reported consistently on the significant influences of learning environment on student engagement. Given little understanding of the engagement of international undergraduate students in China in current literature, it is worth exploring how these students' engagement types, as identified and presented in an engagement typology, are associated with individual demographics and learning environment factors.

## Research Design

### Research Aim and Questions

This research adopted a typological approach and explored the academic engagement of international undergraduate students in Chinese HEIs. Specifically, the following research questions were examined:

1. What is the typology which meaningfully differentiates the participants' academic engagement into distinct types?
2. How are individual student demographic factors associated with the engagement types presented in the engagement typology?
3. How are learning environment factors associated with the engagement types presented in the engagement typology?

### Participants

This research recruited international undergraduate students with no Chinese citizenship studying full-time at 34 Chinese universities in 2016. Given the imbalance of regional development in China's international education, the decision had been made that research participants should be invited from the eastern developed areas, central areas, and the western under-developed areas (Jiang et al., 2015). Within the eastern, central, and western areas, convenience sampling was used for the selection of participating universities. Paper-based questionnaires were printed and posted to the participating universities, together with letters fully explaining the research purposes, clarifying voluntary participation, and expressing thanks. With the help of staff in charge of international student affairs, 3,709 questionnaires were distributed to international undergraduate students in classrooms at the participating universities. These students were invited to fill in the questionnaires within a week at their convenient time. The questionnaires were then retrieved by the staff and posted back to the research team. The research ended with 1,428 valid responses and the response rate was 38.5%. Out of these valid responses, 801 completed all questions required in this study.

Table 1 presents the respondents' characteristics. All these respondents had studied in the host institutions for at least one academic semester when the research was conducted. As presented in Table 1, of the 801 respondents, 411 (51.3%) were male, and 390 (48.7%) were female. One hundred eighty-two (22.7%) were year 1 students, 262 (32.7%) were year 2 students, 127 (15.8%) were in year 3, 140 (17.5%) were in year 4 and 90 (11.2%) were in year 5. Five hundred seventy (71.2%) were from Asia, 185 (23.1%) were from Africa, and 46 (5.7%) were from Europe, America, or Oceania. Six hundred fifty (81.1%) studied in universities located in eastern China, 20 (2.5%) studied in universities located in central China and 131 (16.4%) studied in universities located in western China. The largest proportion of the respondents majored in life sciences and medicine (LSM, *n* = 537, 67.0%), followed by arts, humanities and social sciences (HSS, *n* = 179, 22.3%), and sciences and engineering (SciE, *n* = 85, 10.6%). Slightly over two-thirds of the respondents studied at non-double-first-class universities (*n* = 545, 68.0%), and the rest (*n* = 256, 32.0%) studied at double-first-class universities.

**Table 1:** Questionnaire respondents' characteristics.

**Category**	**Frequency**	**%**
**Gender**		
Male	411	51.3
Female	390	48.7
Total	801	100.0
**Year**		
First	182	22.7
Second	262	32.7
Third	127	15.8
Fourth	140	17.5
Fifth	90	11.2
Total	801	100.0
**Region of origin**		
Asia	570	71.2
Africa	185	23.1
Other (Europe, America, and Oceania)	46	5.7
Total	801	100.0
**Location of host institution**		
Eastern	650	81.1
Central	20	2.5
Western	131	16.4
Total	801	100.0
**Discipline**		
Arts, humanities, and social sciences	179	22.3
Sciences and engineering	85	10.6
Life sciences and medicine	537	67.0
Total	801	100.0
**Institutional academic level**		
Double first-class university	256	32.0
Non-double-first-class university	545	68.0
Total	801	100.0

### Measures

#### Student Engagement

This study used a scale of the Student Experience at Research University International (SERU-I) on student engagement to measure the participants' academic engagement. Although SERU-I was designed to be used in research universities, the following rationales justify our choice of this instrument. First, the SERU-I student engagement scale contains similar items to those in NSSE, and both the SERU-I student engagement scale and NSSE focus equally on students' active engagement in in-class and extracurricular educational activities and on the importance for institutions to support such engagement. Second, although both instruments are used in large-scale surveys, SERU-I involve all year groups of the undergraduate students in the surveyed institutions while NSSE involves first- and last-year undergraduate students only (Klemencic and Chirikov, 2015). Third, SERU-I contains items on campus climate. Compared to NSSE, it focuses more on institutional characteristics and better informs programme reviews (ibid).

Specifically, the SERU-I student engagement scale adopted in this research assesses five dimensions of student engagement: i.e., participation for analytical understanding (AU, 9 items, e.g., *explained methods, ideas or concepts, and used them to solve problems*), meeting academic challenges (MAC, 6 items, e.g., *asked an insightful question in class*), interaction with faculty (IF, 5 items, e.g., *talked with the instructor outside of class about issues and concepts derived from a course*), extracurricular engagement (EE, 5 items, e.g., *worked on class project outside of class*), and lack of engagement (reverse-coded, LE, 4 items, e.g., *skipped class*). The response alternatives were labeled by *never* (scored 1), *rarely* (scored 2), *occasionally* (scored 3), *somewhat often* (scored 4), *often* (scored 5), and *very often* (scored 6).

#### Campus Learning Environment

This research used the SERU-I scale on campus climate to measure two dimensions of international students' perceptions of campus learning environment: i.e., respect for diversity (RD, 8 items, e.g., *Students of my ethnic background are respected on this campus*) and general atmosphere (GA, 5 items, e.g., *My campus is friendly, safe, caring*). Respondents indicated their levels of agreement or disagreement with each item on a scale of 1 to 6, where 1 denotes *strongly disagree* and 6 denotes *strongly agree*.

#### Classroom Learning Environment

To explore the participants' perceptions of classroom learning environment, this research adopted six subscales of the self-developed Chinese University Mathematics Course Experience Questionnaire (UMCEQ, Lu, 2012; Yin and Lu, 2014), complemented with two subscales of the well-established Course Experience Questionnaire (CEQ, McInnis et al., 2001). The six UMCEQ subscales were on teacher support (TS, 6 items, e.g., *Teachers always try their best to help*), peer competition (PC, 4 items, e.g., *We compete with each other in the subject study*), cooperative learning (CL, 6 items, e.g., *I help other students to learn*), innovation in teaching (IT, 4 items, e.g., *Teaching methods are diverse and flexible*), student autonomy (SA, 4 items, e.g., *I have the freedom to choose what I would like to study*) and difficulty of learning (DL, 5 items, e.g., *It is hard to make sense of what is taught in classes*). The two CEQ subscales were on course organization (CO, 5 items, e.g., *My programme is well-organized*) and intellectual stimulation (IS, 9 items, e.g., *Teaching has inspired my enthusiasm for learning*). The responses were measured by 6 levels from 1 (*strongly disagree*) to 6 (*strongly agree*).

A professional translator translated the original UMCEQ items from Chinese into English, and the translated version was cross-checked by a native-English-speaking specialist in Chinese international education. Necessary changes were made to the original SERU-I, UMCEQ, and CEQ items to fit this research's participant population with broader national, disciplinary and linguistic backgrounds. A pilot study was conducted involving 25 senior international students at a Chinese double-first-class university. The information gathered from the pilot study was analyzed by the authors of this article to ensure that the expressions of the final version were clear, and that the content was free from the items which were less relevant to the context of Chinese international student education.

### Data Analysis

Confirmatory factor analysis (CFA), employing AMOS 22.0, was conducted to test the construct validity of the measures. The Cronbach's alpha coefficients were calculated to test the reliability of the measures. Descriptive statistical analysis, using SPSS 22.0, computed means and standard deviations of the responses to the items. Three-factor variance analysis was performed to examine engagement differences by gender, discipline and institutional type.

The research then performed cluster analysis to explore patterns of the participants' responses to the academic engagement scale, conducting an iterative series of *k*-means analysis using SPSS 22.0. Given the exploratory nature of the *k*-means method, the research had set two, three, four and five student-engagement clusters to extract, and by repeating the *k*-means analysis with these pre-set numbers of clusters, different solutions were obtained. The reasons to extract two to five clusters were that two was the minimum number of interpretable clusters, while five was the number of the student engagement dimensions examined in the research. The iterative analysis generated the patterns of academic engagement supporting a three-cluster solution. The results of cluster analysis were firstly evaluated by analysis of variance (ANOVA) and then by discriminant analysis. The discriminant model, analysing the five engagement factors simultaneously, replicated and validated the engagement types generated by the cluster analysis. Multinomial logistic regression analysis was then performed to examine individual and environment factors associated with each engagement type.

## Results

### Scale Construct Validity, Descriptive Statistics, and Three-Factor Variance Analysis

CFA results are presented in Table 2. CFA results confirmed that the scales' structures were acceptable and that the scales provided valid measures of the participants' academic engagement, perceived classroom learning environments, and perceived campus climate.

**Table 2:** CFA results.

**Scale**	**χ2**	***df***	***P***	**RMSEA**	**CFI**	**TLI**
Student engagement	2,412.894	269	*P* < 0.001	0.075	0.889	0.876
Classroom learning environments	5,246.248	830	*P* < 0.001	0.061	0.908	0.899
Campus climate	764.099	64	*P* < 0.001	0.088	0.949	0.938

As shown in Table 3, the mean scores for the five engagement factors were, respectively, 4.85 (LE, reverse-coded), 3.58(AU), 3.53 (EE), 3.49 (MAC), and 2.91 (IF), suggesting inadequate levels of engagement among the respondents. On average, the respondents tended to often attended classes on time or submit assignment before deadlines (LE, reverse-coded), tended to somewhat often engage in academic analysis and comprehension (AU), challenging activities (MAC) or extracurricular learning (EE), and only tended to occasionally interact with faculty (IF). The results also showed that the respondents perceived their academic engagement less positively than their classroom or campus learning environments.

**Table 3:** Reliability, descriptive statistics, and correlations.

	**1**	**2**	**3**	**4**	**5**	**6**	**7**	**8**	**9**	**10**	**11**	**12**	**13**	**14**	**15**
1. SE-AU	(0.917)														
2. SE-MAC	0.517^**^	(0.816)													
3. SE-IF	0.398^**^	0.534^**^	(0.841)												
4. SE-LE (reverse-coded)	0.089^*^	0.123^**^	−0.089^*^	(0.864)											
5. SE-EE	0.474^**^	0.428^**^	0.333^**^	−0.123^**^	(0.803)										
6. CLE-TS	0.283^**^	0.235^**^	0.183^**^	0.171^**^	0.175^**^	(0.934)									
7. CLE-PC	0.251^**^	0.153^**^	0.124^**^	0.152^**^	0.193^**^	0.332^**^	(0.739)								
8. CLE-CL	0.297^**^	0.228^**^	0.133^**^	0.256^**^	0.272^**^	0.440^**^	0.537^**^	(0.909)							
9. CLE-CO	0.310^**^	0.203^**^	0.138^**^	0.193^**^	0.160^**^	0.638^**^	0.333^**^	0.467^**^	(0.919)						
10. CLE-IT	0.196^**^	0.158^**^	0.173^**^	0.077^*^	0.123^**^	0.558^**^	0.412^**^	0.375^**^	0.545^**^	(0.896)					
11. CEL-SA	0.211^**^	0.158^**^	0.173^**^	0.072^*^	0.133^**^	0.479^**^	0.411^**^	0.401^**^	0.592^**^	0.684^**^	(0.832)				
12. CEL-DL	−0.030	0.031	0.085^*^	−0.156^**^	−0.145^**^	0.044	0.160^**^	−0.052	0.060	0.212^**^	0.226^**^	(0.895)			
13. CEL-IS	0.348^**^	0.238^**^	0.174^**^	0.208^**^	0.202^**^	0.686^**^	0.383^**^	0.516^**^	0.863^**^	0.620^**^	0.629^**^	0.081^*^	(0.934)		
14. CC-RD	0.264^**^	0.137^**^	0.125^**^	0.070^*^	0.183^**^	0.454^**^	0.268^**^	0.409^**^	0.490^**^	0.369^**^	0.448^**^	0.030	0.509^**^	(0.929)	
15. CC-GA	0.264^**^	0.140^**^	0.132^**^	0.082^*^	0.117^**^	0.479^**^	0.271^**^	0.366^**^	0.528^**^	0.433^**^	0.516^**^	0.060	0.559^**^	0.555^**^	(0.898)
Mean	3.58	3.49	2.91	4.85	3.53	4.29	4.26	4.43	4.02	3.98	4.02	3.58	4.14	4.21	4.36
SD	1.06	1.11	1.08	0.99	1.07	1.01	0.90	0.87	1.03	1.08	1.05	1.08	0.94	0.96	0.93

* *p < 0.05*,

** *p < 0.01*.

*Each factor's reliability coefficient is presented along the diagonal. SE, Student Engagement; CLE, Classroom Learning Environments; CC, Campus Climate*.

Moreover, Table 3 presents the differences in the correlations between the engagement factors, the classroom environment factors and the campus climate factors, indicating high levels of divergent validity of the factors of the three scales.

Three-factor variance analysis was conducted to examine gender, disciplinary, and institutional differences in relation to the five student engagement factors. Table 4 presents the variance analysis results. As shown in Table 4, gender had a significant main effect on LE (reverse-coded) and EE. Male participants reported significantly lower levels of LE (reverse-coded) and higher levels of EE than female participants.

**Table 4:** Differences in international student engagement by gender, discipline, and institutional type.

	**AU**		**MAC**		**IF**	**LE (reverse-coded)**		**EE**	
	***F***	**Effect**	***F***	**Effect**	***F***	***F***	**Effect**	***F***	**Effect**
Gender	1.277		2.146		3.679	6.686^*^	M < F	8.487^**^	M > F
Discipline	5.206^**^	LSM > HSS	3.789^*^	LSM > HSS SciE > HSS	0.518	10.974^***^	LSM > HSS	10.232^***^	LSM > HSS
Type	2.688		0.055		0.092	0.167		0.831	
Gender × Discipline	0.261		0.490		1.569	1.423		0.565	
Gender × Type	0.627		1.411		0.538	0.171		1.425	
Discipline × Type	1.425		2.924		0.261	0.806		2.045	
Gender × Discipline × Type	2.047		0.027		0.395	1.137		3.058	

* *p < 0.05*,

** *p < 0.01*;

*** *p < 0.001*.

The main effect of discipline was found to be significant on all student engagement factors except IF. Respondents in life sciences and medicine reported significantly higher levels of AU, MAC, LE (reverse-coded), and EE than their counterparts in arts, humanities and social sciences. Respondents in sciences and engineering reported significantly higher levels of MAC than those in arts, humanities and social sciences.

Types of the host institutions presented no significant main effect on the five student engagement factors. None of the five student engagement factors showed significant interaction effects between gender and discipline, between gender and institutional type, between discipline and institutional type, or between gender, discipline and institutional type.

### Cluster Analysis: International Student Engagement Typology

Respectively with two, three, four, or five pre-set clusters to extract, the research repeatedly performed *k*-means cluster analysis. The *t*-test results showed that the two cluster solutions did not present statistically significant variations in LE (reverse-coded) between the two generated clusters. Although the four and five cluster solutions presented statistically significant variations across the five engagement factors, such variations existed only among three out of the four or five generated clusters, suggesting the existence of redundant clusters. In contrast, significant differences existed across the five factors among the three clusters generated by the three-cluster solution, indicating the appropriateness of the three-cluster solution.

**Figure 2:**
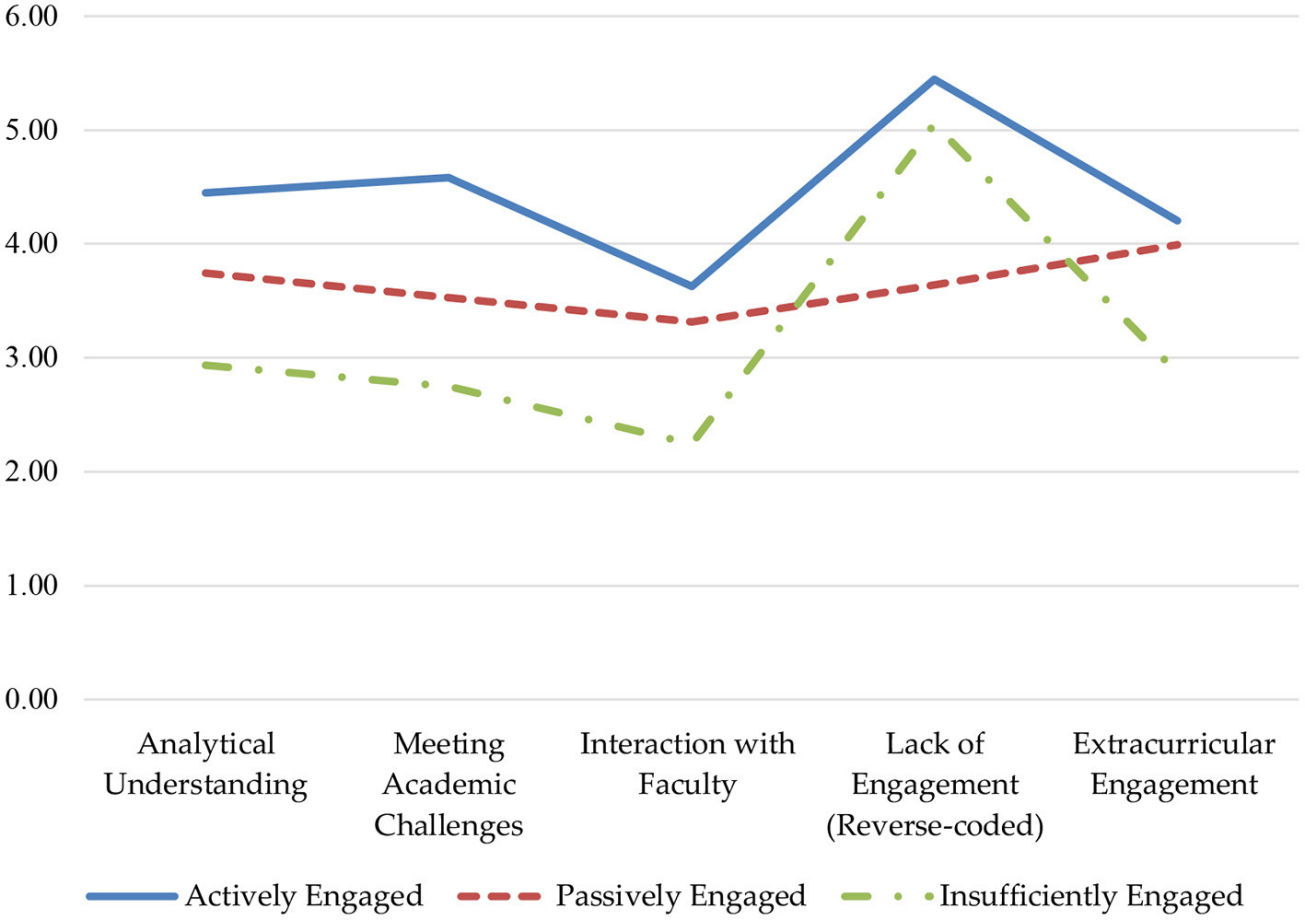
International students' engagement typology.

Hence, the respondents' academic engagement was interpreted as three distinct categories. Figure 2 shows the graphical representation of the cluster profiles. Cluster 1 (*n* = 243, 30.3%) was named an “actively engaged: group, characterized by above-average levels of engagement across the five engagement factors. On average, the respondents in this group often attended classes or submitted course assignment on time, almost often engaged in challenging academic activities, more than somewhat often participated in comprehending materials and learning outside of class, and only tended to somewhat often communicate with faculty.

Cluster 2 (*n* = 184, 23.0%) was named a “passively engaged” group, showing around average levels of engagement. The average passively engaged students tended to attend classes often and submit assignments on time, tended to somewhat often engage in analytical, challenging and extracurricular activities, and only more than occasionally interacted with teachers. It seems that although these students could meet the basic academic requirements on attendance and meeting coursework deadlines, they did not show much effort to engage actively in other productive learning practices.

Cluster 3 (*n* = 374, 46.7%) was labeled as an “insufficiently engaged” group, characterized by below-average levels of engagement, with LE (reverse-coded) as the only exception. Often the average students in this group were able to complete and submit assignments on time and attend classes as required by faculty. However, hardly was there evidence showing their “quality of effort” (Pace, 1990): They only tended to occasionally involve in comprehending, generating and analysing ideas, challenging themselves by contributing to class discussions or making a presentation, and learning outside classes. Slightly more than rarely did they interact with faculty.

The results of one way ANOVA showed that cluster 1 had significantly higher mean scores than cluster 2 across the five engagement factors. Cluster 1 also had significantly higher mean scores than cluster 3 for the factors of AU, MAC, IT, and EE. Cluster 2 presented significantly higher mean scores than cluster 3 for AU, MAC, IT, and EE, but a significantly lower mean score than cluster 3 for LE (reverse-coded). The ANOVA results indicated that across the five engagement factors, the variations within each cluster were significantly lower than the variations between clusters, supporting the distinctiveness of each cluster.

To further test the cluster analysis results, discriminant analysis was performed using the Fisher Linear Discriminant Functions:

$$\begin{array}{l} \text{Active engagement}=-69.963+3.740\;*\text{ AU}+5.063\;*\text{ MAC}\\ \;+4.137\;*\text{ IF}+11.004\;*\text{ LE }\left(\text{reverse}-\text{coded}\right)+5.457\;*\text{ EE}\\ \text{Passive engagement}=-43.832+3.214\;*\text{ AU}+3.623\;*\text{ MAC}\\ \;+3.854\;*\text{ IF}+7.563\;*\text{ LE }\left(\text{reverse}-\text{coded}\right)+5.089\;*\text{ EE}\\ \text{Insufficient engagement}=-42.893+2.252\;*\text{ AU}+2.762\;*\;\\ \text{ MAC}+2.867\;*\text{ IF}+10.168\;*\text{ LE }\left(\text{reverse}-\text{coded}\right)\\ \;+\;4.038\;*\text{ EE} \end{array}$$

For each respondent, the three function values were obtained by substituting the five engagement factors with the scores provided by this respondent on the corresponding factors. The respondent was put into one of the three groups, which presented the highest function value. Table 5 presents the comparison of cluster analysis results and the results of discriminant analysis. As shown in Table 5, the cluster analysis placed 30.3% of the respondents into the active engagement cluster, 23.0% into the passive engagement cluster and another 46.7% into the insufficient engagement cluster. The discriminant analysis results showed 97.5% correctness for the active engagement cluster, 91.3% correctness for the passive engagement cluster and 92.8% correctness for the insufficient engagement cluster.

**Table 5:** Comparison between cluster analysis and discriminant analysis results.

		**Cluster analysis**			**Total**
		**Active engagement**	**Passive engagement**	**Insufficient engagement**	
Discriminant analysis	Active engagement	237	4	7	248
	Passive engagement	6	168	20	194
	Insufficient engagement	0	12	347	359
Total		243	184	374	801

### Multinomial Logistic Regression: Factors Associated With International Student Engagement Types

Multinomial logistic regression analysis was conducted to explore whether and how the passive and insufficient types of engagement, using active engagement as the reference group, were associated with the given individual and learning environment factors. Results are presented in Table 6. The analysis used engagement types as the dependent variables, and student demographics and learning environment factors as independent variables. The value of initial intercept-only −2ll with no independent variables was 1,689.295. The −2ll value for the logistic regression model including the independent variables were 1,470.874 (*p* < 0.001). The difference between these two −2ll values was statistically significant, indicating a satisfying goodness-of-fit of the regression model (Menard, 2002). It is worth noting that the regression analyzes also computed the interaction effects on the three engagement types between gender and discipline, between gender and institution type, between discipline and institution type, or between gender, discipline and institution type. These results are not presented in this article, as no significant interaction effects were found.

**Table 6:** Multinomial logistic regression: factors predicting engagement types.

**Independent variable**	**Dependent variable:engagement type^a^**			
	**Passive engagement**		**Insufficient engagement**	
	***B***	**Exp(B)**	***B***	**Exp(B)**
**Gender (male as reference)**				
Female	−0.298	0.742	0.652^**^	1.919
**Year (year 3, 4 & 5 as reference)**				
Year 1 & 2	0.156	0.856	−0.106	0.899
**Region of origin (other continents as reference)**				
Asia	−0.206	1.229	−0.321	0.725
Africa	−0.555	0.574	−0.722	0.486
**Discipline (LSM as reference)**				
HSS	1.161^***^	3.194	0.759^**^	2.137
SciE	1.140^**^	3.127	0.729^*^	2.074
**Institution type (Double-first-class university as reference)**				
Non-double-first-class university	0.614^*^	1.847	0.777^***^	2.175
**CLE**				
TS	−0.105	0.900	−0.285^*^	0.752
PC	−0.245	0.783	−0.263^*^	0.769
CL	−0.751^***^	0.472	−0.529^**^	0.589
CO	0.011	1.011	0.229	1.257
TI	0.178	1.195	0.166	1.180
SA	0.211	1.235	0.040	1.041
DL	0.205	1.228	0.116	1.123
IS	−0.609^*^	0.544	−0.627^**^	0.534
**CC**				
RD	0.275	1.316	0.021	1.021
RD	−0.123	0.885	−0.074	0.929
Cox & Snell	0.239			
Nagelkerke	0.272			
McFadden	0.129			

* *p < 0.05*;

** *p < 0.01*;

*** *p < 0.001*.

a *Active engagement as the reference group. HSS, humanities and social sciences; SciE, sciences and engineering; LSM, life sciences and medicine; CLE, Classroom Learning Environments; CC, Campus Climate. Grade level refers to the grade in which an individual participant was studying in when the research was conducted*.

#### Passive Engagement

As shown in Table 6, in comparison to the respondents in LSM, those in HSS and SciE were, respectively, 2.194 and 2.127 times more likely to be passively, rather than actively, engaged in learning. In comparison to the respondents in double-first-class universities, those in non-double-first-class institutions were 0.847 times more likely to be passively, rather than actively, engaged in learning. With respect to the eight classroom learning environment factors, PC and IS significantly predicted the reduced probability of passive engagement. The respondents who perceived more positively the factors of CL and IS were significantly more likely to be actively, rather than passively, engaged in learning.

#### Insufficient Engagement

As Table 6 shows, in comparison with the male respondents, the female respondents was 0.919 times more likely to demonstrate insufficient, rather than active, engagement in learning. In comparison to LSM students, those in HSS and in SciE were, respectively, 1.137 and 1.074 times more likely to be insufficiently engaged. In comparison to respondents in double-first-class universities, their peers in non-double-first-class institutions were 1.175 times more likely to be insufficiently engaged in learning. With respect to classroom learning environment, the respondents who perceived more positively the factors of TS, PC, CL, and IS were significantly more likely to be actively, rather than insufficiently, engaged in learning.

Table 7 presents student demographic distributions among the three engagement types. As shown in Table 7, significant differences in distribution were found between gender groups. More than half of the actively engaged respondents (54.7%) and the passively engaged respondents (66.3%) were male students, while the highest percentage of the insufficiently engaged respondents (58.3%) were female students. It should be noted that of the whole sample, the male gender group constituted a slightly higher proportion than the female gender group. After accounting for this imbalance, it was shown that the percentage of actively engaged students was higher among males than females, and the percentage of insufficiently engaged students were higher among females than males.

**Table 7:** Distribution across the three types of engagement.

**Variable**	**Active engagement**			**Passive engagement**			**Insufficiently engagement**			**χ^2^**
	**Number**	**%**	**Ratio^a^**	**Number**	**%**	**Ratio^a^**	**Number**	**%**	**Ratio^a^**	
**Gender**										
Male	133	54.7	1.123	122	66.3	1.361	156	41.7	0.856	31.491^***^
Female	110	45.3	0.883	62	33.7	0.657	218	58.3	1.136	
Total	243	30.3		184	23.0		374	46.7		
**Grade level**										
Year 1 & 2	139	57.2	1.027	105	57.1	1.025	202	54.0	0.969	0.793
Year 3 & 4	104	42.8	0.966	79	42.9	0.968	172	46.0	1.038	
Total	243	30.3		184	23.0		374	46.7		
**Region of origin**										
Asia	154	63.4	0.890	146	79.3	1.114	270	72.2	1.014	31.067^***^
Africa	83	34.2	1.481	27	14.7	0.636	75	20.1	0.870	
Europe, America and Oceania	6	2.5	0.439	11	6.0	1.053	29	7.8	1.368	
Total	243	30.3		184	23.0		374	46.7		
**Discipline**										
HSS	30	12.3	0.552	56	30.4	1.363	93	24.9	1.117	31.102^***^
SciE	23	9.5	0.896	28	15.2	1.434	34	9.1	0.858	
LSM	190	78.2	1.167	100	54.3	0.810	247	66.0	0.985	
Total	243	30.3		184	23.0		374	46.7		
**Institution type**										
Bouble-first-class	85	35.0	1.094	64	34.8	1.088	107	28.6	0.894	3.623
Non-double-first-class	158	65.0	0.956	120	65.2	0.959	267	71.4	1.050	
Total	243	30.3		184	23.0		374	46.7		

a *Results of calculations which divide the proportion of each variable group in an engagement type by the proportion of the same variable group in the whole sample. For instance, the proportion of the male respondents among the active engagement type is 54.7%, the proportion of the male gender group among the whole sample of students is 48.7%, and the ratio of the two is 1.123*.

*** *P < 0.001*.

*HSS, humanities and social sciences; SciE, sciences and engineering; LSM, life sciences and medicine*.

Significant distribution differences were also noted between the respondents with different regions of origins. Asian students accounted, respectively, for 63.4, 79.3, and 72.2% of the total actively engaged, passively engaged, and insufficiently engaged respondents. Those from Europe, America, and Oceania accounted, respectively, for 2.5, 6.0, 7.8% of the total actively engaged group, the passively engaged group and the insufficiently engaged group. Besides, in this research, the Asian respondent group constituted a higher proportion than the African group, or the group of respondents from Europe, America and Oceania. After accounting for this imbalance, it was shown that the percentage of actively engaged students were the highest among African respondents, the percentage of passively engaged students was the highest among Asian students, and the percentage of insufficiently engaged learners was the highest among students from Europe, America and Oceania.

In addition, significant differences in distribution existed between the respondents studying in different disciplines. LSM students accounted, respectively, for 78.2, 54.3, and 66.0% of the total actively engaged, passively engaged and insufficiently engaged respondents. HSS students accounted, respectively, for 12.3, 30.4, 24.9% of the total actively engaged group, the passively engaged group and the low engaged group. Besides, in this research, the LSM respondent group constituted a higher proportion than the HSS respondent group or the SciE respondent group. After adjusting this imbalance, the percentage of actively engaged students were still the highest among LSM respondents, the percentage of passively engaged students were the highest among SciE respondents, and the percentage of insufficiently engaged students was the highest among HSS students.

No significant differences in distribution were found between the respondents with different grade levels, or between the respondents studying at double-first-class universities and those at non-double-first-class institutions.

## Discussion

### A Typology of International Student Engagement

The empirical analysis identified distinct groups among international undergraduate students in Chinese HEIs with respect to their engagement in meaningful educational activities. The three-category typology, developed using *k*-means cluster analysis and validated by discriminant analysis, classified the respondents' engagements as active, passive or insufficient. These findings confirmed the usefulness of a typological approach in understanding international student engagement at Chinese universities and, in line with the previous research, revealed that the engagement types presented in the typology were related to international students' demographic backgrounds and their perceived learning environment factors.

The typological results pointed to the less than satisfactory levels of academic engagement among the research respondents. The absolute majority of the respondents displayed passive or insufficient engagement. Those reporting passive engagement, which accounted for 23.0% of all respondents, were less likely to be actively involved in knowledge construction activities. The percentage of the respondents reporting insufficient engagement was as high as 46.7%. These students only managed to attend classes or turn in assignments on time. Rarely did they participate in the activities and events that would lead to productive learning. These results highlight inadequacies in China's international undergraduate education if it is agreed that the quality and the sustainability of its international education should be centered around international students and their learning.

### Demographic Factors Associated With Engagement Types

The current research examined the association between individual demographics and the engagement types presented in the engagement typology. This research revealed consistent patterns with respect to gender groups. Three-factor variance analysis showed that male respondents reported lower levels of LE (reverse-coded) and higher levels of EE than female respondents, indicating that male respondents put greater effort in meeting basic course requirements and were more actively engaged in out-of-class activities. Multinomial logistic regression analysis, after controlling for other variables, showed that female respondents were more likely to be insufficiently engaged. In terms of gender distribution across the three engagement types, most of the active learners were male students, while most of the insufficiently engaged learners were female students. The results contradicted the findings of the research conducted in both Western (Sax and Harper, 2005; Pryor et al., 2007) and Chinese contexts (Shi et al., 2011). To explain the results, we would like to highlight the complexity of international student engagement in intercultural learning, in which academic difficulties are often intertwined with and exacerbated by language barriers, communication incompetency and local intolerant attitudes (Mann, 2001; Tian and Lowe, 2009, 2018). These intercultural difficulties may have more greatly impacted the engagement of international female students, as females were found to be more sensitive to negative learning experiences than their male peers (Sax and Harper, 2005).

In addition, the findings of this research showed that, when considering the proportions of the three groups categorized by regions of origin in the whole sample, the percentage of actively engaged learners was the highest among the African respondent group. The literature on international students has drawn on cultural distance to explain the variations of different national groups in intercultural adaptation, with larger distances between the host and home cultures related to poorer adaptation (Suanet and van de Vijver, 2009). The greater likelihood of active learning engagement among African respondents, rather than Asian respondents, suggests an oversimplification in resorting to cultural distances to explain acculturative behaviors. Factors other than cultural differences may also have affected the engagement of the respondents with different regions of origin, such as individual students' socioeconomic status (Yu et al., 2014), proficiency in both Chinese and English (Wen et al., 2018), reasons for and expectations of learning in China (Tian and Lowe, 2018), and plans after graduation. Future research is needed to further explore the issue.

With respect to disciplinary differences, the three-factor variance analysis revealed that LSM respondents reported significantly higher levels of active engagement in AU, MAC, LE (reverse-coded), and EE than those in HSS disciplines, and SciE students reported significantly higher levels of MAC than those in HSS disciplines. Multinomial logistic regression analysis revealed that HSS and SciE respondents were more likely than the LSM respondents to be passively or insufficiently engaged in learning. Analysis of the disciplinary distribution across the three types of engagement, when considering the group proportions in the whole sample, showed that the percentage of actively engaged learners was the highest among LSM majors. These findings displayed significant differences from those of studies conducted in American HEIs (Umbach and Wawrzynski, 2005; Matthews et al., 2011) and studies on domestic students at Chinese universities (Shi and Wen, 2012), revealing the distinct features of academic engagement among international undergraduates in China.

The higher levels of active engagement among LSM students may be explained by the comparatively extended history of the curricular development and quality control schemes in medicine. Medical programmes were among the first English-medium undergraduate programmes that Chinese universities offered to international students. In China, *the Quality Control Standards for English-Medium International Student Undergraduate Education in Medicine* was released in 2007 (Ministry of Education, China, 2007), 11 years earlier than the release of *the Quality Standards for International Student Higher Education in China (trial version)*, the first national quality control policy for general programmes in international degree education (Ministry of Education, China, 2018).

The less active engagement among SciE respondents requires attention. China's great investments for world-leading excellence may have resulted in significant improvements in research productivity in sciences and engineering disciplines (Institute of Scientific and Technical Information of China, 2019) and the high rankings of these disciplines in international league tables (Shanghai ranking, 2019b). The national strategic initiatives, however, have not generated significant effects on academic engagement of international undergraduates in these disciplines.

Moreover, with respect to institutional types, the findings of this research are also worth noting. Multinomial logistic regression analysis showed that, when controlling for other variables, the respondents in non-double-first-class institutions were more likely to be passively or insufficiently engaged than those in double-first-class institutions. Yet three-factor variance analysis did not reveal significant main effects of institutional type on the five engagement factors; nor did any engagement factor present significant interaction effects between gender, discipline and institutional type. Furthermore, no significant differences in distribution of the three engagement types were found between the respondents studying at non-double-first-class universities and those at double-first-class universities. These mixed findings require cautious interpretation: The results apparently support the claim that national leading double-first-class universities were in better conditions to support students' learning (Shi et al., 2011). Nevertheless, the “better condition” may be largely restricted to the improved “hardware,” i.e., resources and facilities, rather than the improved quality of teaching or course management (Shi et al., 2011). Another possible explanation for the findings may be heavy research pressure faced by the faculty at double-first-class universities, which may have limited their time and energy to support international student engagement (Huang et al., 2021, p. 125).

### Learning Environment Factors Associated With Engagement Types

The research analyzed the association between learning environments and the engagement types presented in the engagement typology. It revealed that after controlling for other variables, the four classroom learning environment factors, which had significant effects, could be grouped into two categories. The first group included CL and IS, which were associated with reduced likelihoods of both passive and insufficient engagement. The second group included TS and PC, which were associated with reduced likelihood of insufficient engagement. The results are in line with a large number of studies that documented the positive impacts of cooperative learning, inspiring teaching, teacher support, and peer competition on student engagement (Ahlfeldt et al., 2005; Umbach and Wawrzynski, 2005). It should be noted that the findings on the positive impact of cooperative learning differed from the previous observations of Chinese undergraduate classrooms where students were often passive learners (Yin and Wang, 2016), indicating the importance of avoiding essentialist interpretations of China's learning culture in its increasingly internationalized HEIs.

This research revealed no significant effects of campus climate factors on international student engagement after controlling for other variables. This finding is not surprising, as host teachers and peer students are those with whom international students have direct contact on a daily basis. This result reflects the greater responsibilities that teaching faculty play in encouraging and supporting academic engagement of international students, for whom university culture may be “foreign and at times alienating and uninviting” (Krause, 2005, p. 9; Tian and Lowe, 2018).

## Limitations

The research has the following limitations. First, the sample may not reflect the demographic distributions of the international undergraduate student population in China. Particularly, this research did not collect information regarding whether a participant was self-funded or sponsored by a scholarship. Future research could explore how funding variations are associated with engagement types of international undergraduate students in Chinese HEIs. Second, the research developed a typology based on the analysis of engagement features over five dimensions. Other ways to measure engagement may shed new light on engagement types of international students in China. Third, this research discussed international student engagement by regions of origin rather than specific home countries. This is because the number of respondents from one home country could be small to allow for more varied statistical analysis. We acknowledge the cultural and linguistic differences existing within geographical regions and recommend that future engagement research addresses such differences. Moreover, this research suggested important individual and environmental factors associated with international student engagement types. More sensitive research combining a longitudinal design with in-depth qualitative methods are needed to investigate further individual and contextual variables and their impact on international student engagement. An additional limitation is that the current study used self-reported data. Although the validity of self-reported data has been well-argued (Kuh, 2009a), such data may not reflect the actuality of learning and teaching.

## Conclusions and Implications

Rapid international student enrollment expansion in China has called for quality assessment and monitoring of international education among Chinese HEIs. Drawing on a survey involving 801 international undergraduate students at 34 Chinese HEIs, this research responded to the need by developing an international student engagement typology and examining individual and learning environment factors associated with the engagement types presented in the typology. As one of the first attempts to discuss and assess Chinese international education quality from the perspective of international students, this research deepens understandings of the potentials and limitations in the current development of China's international undergraduate education. Although focusing on China, it holds implications for practice and policy in a broader range of contexts.

Specifically, the identification of the three-category typology of international student engagement, as validated by the discriminant analysis, is important. It differentiates international student engagement into the active, passive and insufficient types. The typology indicates that international student engagement, conceptually, does not involve the dichotomous patterns of either being engaged or disengaged, but rather lies on a continuum between engagement and disengagement, and that each engagement style comprises multiple dimensions in which the same student may exhibit different levels of involvement.

Empirically, the research revealed that nearly half of the respondents were insufficiently engaged, and another 23% were passively engaged in learning. On average, the levels of the respondents' engagement across the five dimensions were less than satisfactory. While fully acknowledging the difficulties and challenges posed by intercultural learning, international students are suggested to exert agential power and put greater efforts into meeting course expectations, seeking academic challenges, improving communication with faculty, and participating proactively both in- and outside of classrooms. The specific findings of this research can also be used by individual international students to monitor personal engagement so as to maximize their learning outcomes in Chinese higher education.

In addition, the research revealed the variations in the respondents' engagement by gender, region of origin, discipline and institution type. The results highlight the problematic intentions that come with viewing international students as a homogeneous group. While it is important to further our understandings of the shared needs, concerns and difficulties of international students, it should be well-recognized that students are different, and variations in personal backgrounds can affect their involvements in learning. The findings necessitate regular assessments across group demographics to better understand the engagement of the diverse international student population.

Notably, the high proportion of passively and insufficiently engaged respondents triggers an alarming call for quality control in Chinese international education. To retain and further enhance China's attractiveness among globally mobile students, it is urgent that evidence-based measures are taken to intervene and improve international students' levels of engagement. This research confirmed that supportive learning environments, which provided ample opportunities for analytical understanding, academic challenges, interactions with faculty and extracurricular engagement, were crucial for active engagement. Particularly, it revealed that peer cooperation and intellectual stimulation were significantly and positively associated with the reduced possibility of passive and insufficient engagement. Administrators and faculty are suggested to adopt innovative pedagogy to encourage cooperative learning among international students and integrate inspiring learning opportunities to support their development. Chinese national double-first-class universities and in particular sciences and engineering disciplines in these universities, given the resource advantages they enjoy, are expected to play a leading role in creating an environment where international students are challenged to extend their “quality of effort” (Pace, 1990) in learning, while feeling well-supported and welcomed into the “new” academic community.

## Data Availability Statement

The raw data supporting the conclusions of this article will be made available by the authors, without undue reservation.

## Ethics Statement

Ethical review and approval was not required for the study on human participants in accordance with the local legislation and institutional requirements. The patients/participants provided their written informed consent to participate in this study.

## Author Contributions

MT and GL contributed equally to the design of the research, the collection, analyzes, or interpretation of the research data, and the writing of the paper. LL contributed to the initial data analysis. HY reviewed the manuscript. All authors have read and agreed to the published version of the manuscript.

## Conflict of Interest

The authors declare that the research was conducted in the absence of any commercial or financial relationships that could be construed as a potential conflict of interest.

## Footnotes

^1^CNKI provides comprehensive online academic resources in the Chinese language, including over 7,500 journals, doctoral and Master's dissertations of over 650 Chinese HEIs, over 500 newspapers, 3,200 types of yearbooks and 3600 types of teaching references (cnki.net, 2020). Compared to another two major Chinese academic databases, *Wanfang* data and *VIP*, CNKI covers most comprehensively Chinese domestic academic journals and the academic journals it covers are of better quality (Yang, 2020). Both *Wanfang* data and *VIP* have a focus on Chinese academic resources in science and technology (ibid).
